# Vitamin A- and C-rich *Pouteria camito* fruit derived superparamagnetic nanoparticles synthesis, characterization, and their cytotoxicity

**DOI:** 10.4314/ahs.v22i1.78

**Published:** 2022-03

**Authors:** Chinnadurai Veeramani, Ahmed S El Newehy, Mohammed A Alsaif, Khalid S Al-Numair

**Affiliations:** Department of Community Health Sciences, College of Applied Medical Sciences, King Saud University, P.O. Box 10219, Riyadh 11433, Saudi Arabia

**Keywords:** Fruit, Pouteria caimito, ferric chloride, superparamagnetic nanoparticles, cytotoxicity

## Abstract

**Background:**

Recently, green nanoparticles are gaining importance in drug development because of their lower toxicity, sustainability, cost effectiveness, simplicity, and ecofriendly nature compared with toxic chemicals.

**Objective:**

In this study, we developed a nontoxic method for synthesizing iron oxide nanoparticles by using the fruit of Pouteria caimito that is rich in vitamin A and C and evaluated their cytotoxicity.

**Methods:**

Pouteria caimito fruit¬-derived superparamagnetic nanoparticles (PCSNs) were characterized using physical and chemical methods, and their cytotoxicity was examined using the 3-(4, 5-dimethylthiazol-2-yl)-2–5-diphenyltetrazolium bromide (MTT) assay.

**Results:**

Ultraviolet-visible spectroscopy (UV-Vis spectro) analysis of PSNs showed a peak at 277 nm. Transmission electron microscopy (TEM) findings showed that PSNs exhibited a nanorod shape with their sizes ranging from 9.41 nm to 16.96 nm (average size: 13.08 nm). The findings of dynamic light scattering (DLS) indicated that the particle size was 186. 6–847.3 d.nm with an average of 367.5 d.nm. The Zeta potential analysis indicated that PSNs exhibited uniform surface charge distribution, and their surface charge was equal to −13.7 mV. Fourier-transform infrared spectroscopy (FTIR) analysis showed that PSNs exhibited bands at 3412, 1629, 1384, 1075, 818, 697, and 471 cm−1. Energy-dispersive X-ray spectroscopy (EDX) results showed that iron was the major element present in PCSNs, followed by other biomolecules such as C, O, and Cl, indicating the production of iron oxide nanoparticles.

**Conclusion:**

The Pouteria caimito fruit that possesses strong oxidizing and nontoxic properties can be a potentially attractive source for the production of iron oxide nanoparticles. Moreover, the cytotoxicity assay results revealed that iron oxide nanoparticles synthesized using the Pouteria caimito fruit extract derived can be used for targeting cancer cells and treating other diseases because of their nontoxic nature. These nanoparticles can be used for the treatment of cancer and other diseases in the future.

## Background

The synthesis of nanoscale materials is currently a rapidly emerging field in nanoscience and nanotechnology because of their particular characteristics such as shape, size, and distribution^1^,^2^. Numerous chemical and physical techniques are available for synthesizing nanoparticles; however, these techniques have drawbacks, particularly in biomedical applications, and pollute the environment. Hence, alternative techniques have been recently developed for synthesizing nanoparticles associated with plants, fruits, fungi, bacteria, and algae that have considerable applications in biomedical and pharmaceutical fields^3^–^5^. Nanoparticles have been synthesized using numerous precursors including silver, gold, nickel, iron, zinc, and copper because of their various benefits^6^, ^7^. Iron oxide is a naturally available mineral compound that possesses highly reactive surfaces and a large specific surface area. Iron oxides having unique physical and chemical characteristics and various biomedical applications. Nanoparticles synthesized using a natural plant source are gaining importance because they contain plant-derived biocompatible molecules that are not involved or damaged during chemical and physical reactions occurring in nanoparticle synthesis^6^. However, plant-derived biocompatible molecules are more affordable and gaining increased importance compared with other biomolecules such as peptides, protein, DNA, and enzymes because they are nonhazardous, cost effective, and ecofriendly; serve as capping and stabilizing agents; and exhibit stability against environmental factors such as pH, temperature, and salinity^4^, ^6^.

Pouteria caimito is a common fruit belonging to the Sapotaceae family and is widely available in the international market. Pouteria caimito has been used as a traditional medicine for stomach-related problems such as pain and digestion. The fruit of Pouteria caimito contains nutritional ingredients such as proteins, carbohydrates, fats, fiber, riboflavin, vitamin A, niacin, vitamin C, thiamine, Ca, and Fe 8. Moreover, Pouteria caimito possesses anti-inflammatory and free radical-scavenging properties^9^. Our previous study reported that Pouteria caimito can be used for synthesizing silver nanoparticles and core-shell nanospheres and can be potentially used for treating cancer^10^. However, superparamagnetic iron nanoparticles have not yet been synthesized using the fruit of Pouteria caimito, and their cellular cytotoxicity has not yet been assessed. Cytotoxicity studies involve the determination of the cytotoxic effects of different drugs and pesticides including protein synthesis prevention, cell membrane destruction, and irreversible receptor binding. Low-cost and reliable cytotoxicity and cell viability assays are currently used in numerous fields such as toxicology and pharmacology. MTT assay is the colorimetric assay mostly used to evaluate the cytotoxicity of new drugs or cell viability 11. Hence, in this study, we synthesized superparamagnetic iron nanoparticles from the fruit extract of P. caimito and evaluated their cytotoxic effects on 3T3 and MCF-7 cells.

## Methods

### Materials

Ferric chloride and others chemicals were obtained from Sigma-Aldrich, St. Louis, Missouri, United States. Fresh fruit of Pouteria caimito was collected from the shop at Riyadh, Saudi Arabia.

### *Pouteria caimito* fruit aqueous extraction

Fresh fruit of Pouteria caimito cut into small sections about 0.2 to 0.4 inches sizes and shade dried for 3 days. The dried fruit sections were made powder form by using homely mixer speed 15000 rpm for 3 mins. The aqueous extract was prepared by 5 g of power was immersed with 250 mL of distilled water for 24 hrs. The extract was filtered after 24 hrs by whatman filter paper. This fresh Pouteria caimito fruit extract was used for nanoparticle synthesis.

### Synthesis of PCSNs

PCSNs was produced by ecofriendly method. 1.10 g of ferric chloride (FeCl3.6H2O) was dissolved with 100 mL of deionized water and this was heated 80 °C for 10 mins under warm stirring. Subsequently, 20 mL of Pouteria caimito fruit extract was added slowly followed by adding 15 mL sodium hydroxide under warm stirring by magnetic stirrer. The appeared black color had designated the nanoparticles production. The block mixture was kept for cool 1 hr and discorded the clear supernatant. The residue of block mixture was washed and centrifuged (10000 rpm for 15 mins) three times by using deionized water. After washed the mixture was kept for air oven 39 °C for 12 hrs and made dried powder form. The powder form of PCSNs was used for physical and chemical confirmation studies and also evaluate their biological applications.

### PCSNs characterization

The visual characterization of biotic superparamagnetic nanoparticles was inspected by UV-Vis- spectro analysis. The functional groups of PCSNs was examined by using a FT-IR spectroscopy. Elements analysis was carried out in biotic superparamagnetic nanoparticles by EDX. Morphological and size distributions of PCSNs were analyzed by TEM, DLS and SEM methods 12.

### Cytotoxicity measurement by MTT method

MTT assay is extensively used by scientist to evaluate the cells metabolic activities. The cytotoxic effect of PSNs was evaluated by MTT assay on 3T3 and MCF-7 cells. A density of 104 cells with 100 µl volume of 3T3 and MCF-7 cells were kept in each well of 96 well plates at different time interval measurements such as 24, 48 and 72 hrs. The cells of 96 well plates were incubated at 37 °C with 5% CO2 for 24 hrs. After incubated the cells were attached in bottom of the wells and changed the new medium. The various concentrations of (50 µg/ml, 100 µg/ml, 1 mg/ml, 5 mg/ml, 10 mg/ml and 20 mg/ml) of PSNs dissolved with in culture medium then it was added into wells individually since 5 repetitions for each concentration. PBS was added in control group and it was used as a witness of control group instead of nanoparticles. MTT solution (10 µl (5 mg/ml)) was incorporated into each well and then wells were incubated at 37 °C for 4 h. The formazan crystals have formed after 4 hrs incubation then the medium was removed including MTT. The DMSO (100 µl) was added in each well and dissolved by using shaker. The absorption of formazan crystals solution was read at 570 nm by plate reader device.

### Statistical method

Results are represented as the average of 3 readings (Mean average ± Standard error). A statistical study of this outcomes was evaluated by two-way ANOVA of Tukey's tests. P value ≤ 0.05 was specified as in statistically significantly of results.

## Results

### UV-Vis spectrum of PCSNs

**Figure 1:**
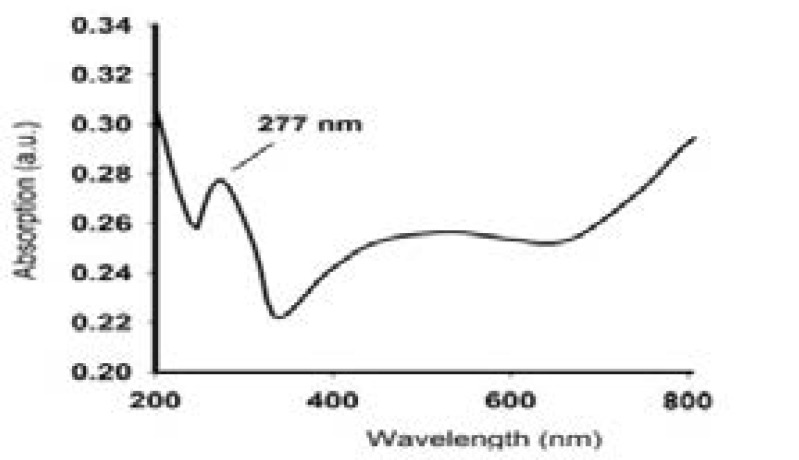
UV absorption spectroscopic image of synthesized biotic superparamagnetic nanoparticles from *Pouteria caimito* fruit aqueous extract

Fig. 1. shows the UV-Vis spectrum of PCSNs. Generally, the nanoparticle size, and dispersal were evaluated primarily by UV-light absorption spectrum at various wavelengths. However, our study also determined UV-Vis absorbance spectrum of PCSNs at different wavelengths and the result displayed at 277 nm.

### TEM spectrum of PCSNs

**Figure 2:**
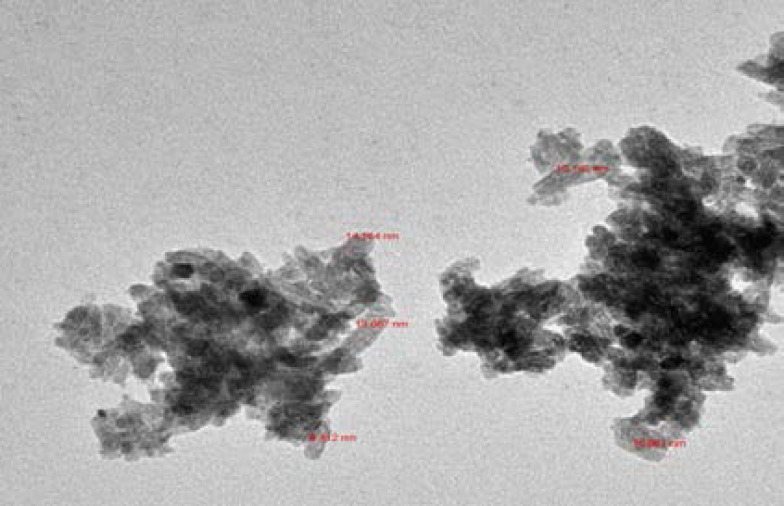
TEM image of synthesized biotic superparamagnetic nanoparticles from *Pouteria caimito* fruit aqueous extract.

Fig. 2. displays the TEM spectrum of PCSNs. In our study, the PCSNs was found in nanorod shape with the size ranged from 9.41 nm to 16.96 nm (average size: 13.08 nm).

### EDX spectrum of PCSNs

**Figure 3:**
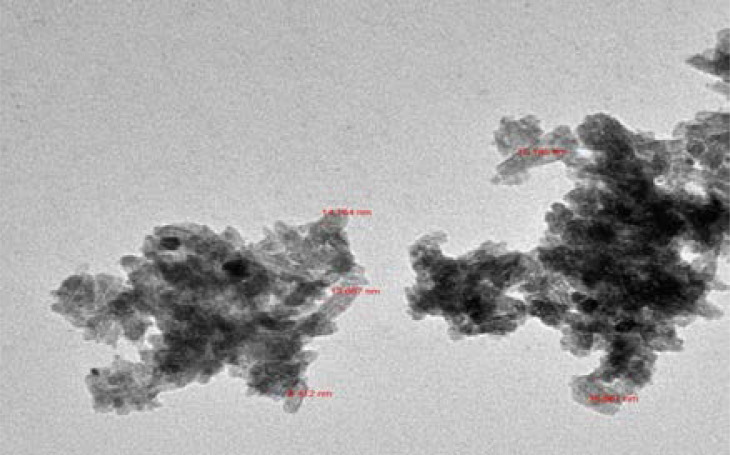
EDX image of synthesized biotic superparamagnetic nanoparticles from *Pouteria caimito* fruit aqueous extract.

Fig.3. displays the EDX spectrum of PCSNs and represents the presented biomolecules. Our spectrum confirmed that the PCSNs production by the presented biomolecules were overlapped with the iron nanoparticles since iron was the chief element. Further, others biomolecules also present such as C, O, and Cl, which clearly indicated that the iron oxide nanoparticles productions.

### DLS analysis of PCSNs

**Figure 4:**
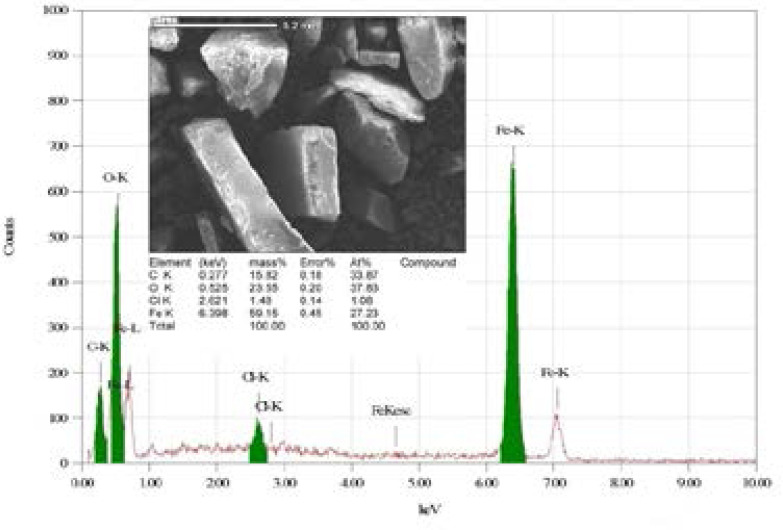
Zeta potential and size distribution measurement of synthesized biotic superparamagnetic nanoparticles from *Pouteria caimito* fruit aqueous extract by

Fig. 4. Shows the PCSNs size distribution and zeta potential by DLS analysis. In this study, the PCSNs size distribution was observed at 186. 6–847.3 d.nm with the average of 367.5 d.nm. Zeta potential analysis of PCSNs showed the uniform surface charge distribution and designates the surface charge is equal to −13.7 mV. FTIR spectrum of PCSNs

**Figure 5:**
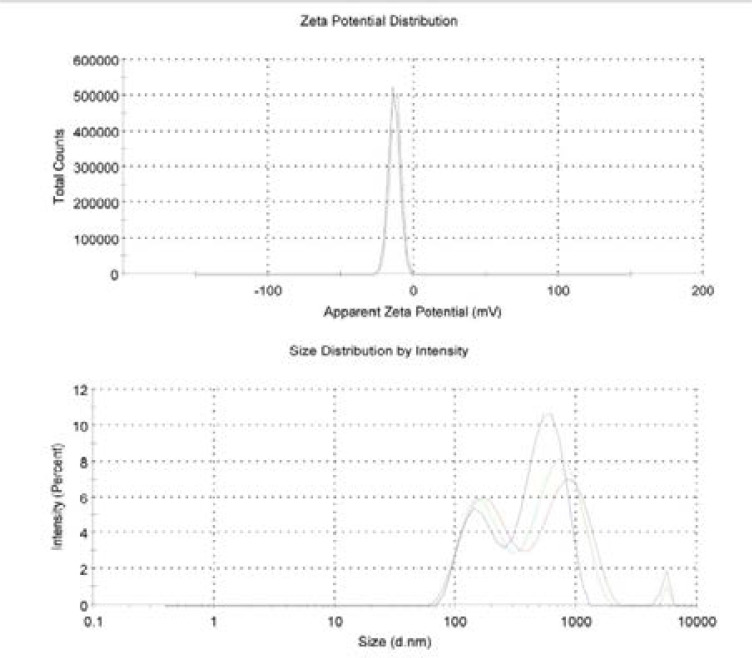
FTIR image of synthesized biotic superparamagnetic nanoparticles from *Pouteria caimito* fruit aqueous extract

Fig. 5. Displays the FTIR spectrum of PCSNs. The bands at 3412 cm−1 is indicated aliphatic and aromatic N-H stretching which indication the amines. The band 1629 cm−1 is associated with the Carbonyl (C=C) group. Peaks at 697 cm−1 and 471 cm−1 related to the Fe-O stretching. The band at 697 cm−1 is allied with Fe-O-H stretching. The bands 1075 and 1384 cm−1 is indicated the covalent attachment to nanoparticle or ester. The band 818 cm−1 is assigned with the C-C stretching of alkanes.

### Cytotoxic assay of PCSNs

**Fig. 6:**
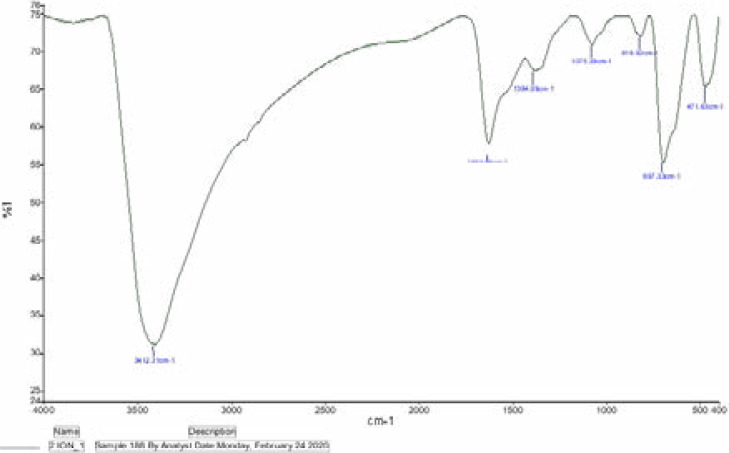
Examination of Viability of MCF-7 cells on treating with biotic superparamagnetic nanoparticles after 24, 48 and 72 h respectively Data are mentioned as average of 3 reading (Mean ± SE) *symbol is mentioned the statistically significant in concentration dependent manner P≤0.05 (DMRT)

**Fig. 7:**
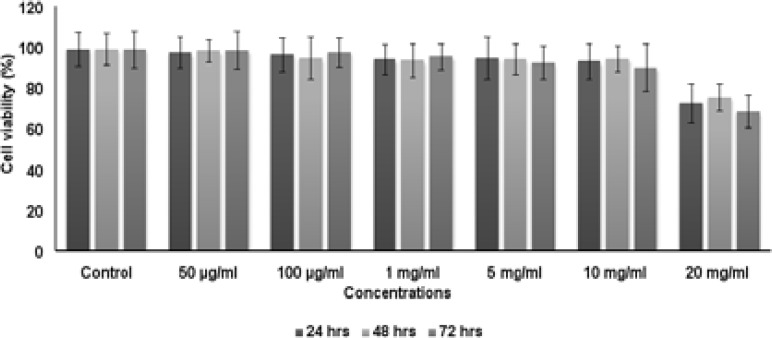
Examination of Viability of 3T3 cells on treating with biotic super paramagnetic nanoparticles after 24, 48 and 72 h respectively Data are mentioned as average of 3 reading (Mean ± SE) *symbol is mentioned the statistically significant in concentration dependent manner P≤0.05 (DMRT)

Fig.6 and 7 show the cytotoxicity assessment of PCSNs on MCF-7 and 3T3b cells. 50 µg/ml, 100 µg/ml, 1 mg/ml, 5 mg/ml, 10 mg/ml and 20 mg/ml of PCSNs were used to test cytotoxicity assays and the results observed that the minimal toxicity was produced on both cell lines with a higher concentration only. This minimal toxicity on higher dose is most advantages for natural cells as a positive property in reducing side effects.

## Discussion

Recently, an environmentally friendly and nontoxic method has been developed for synthesizing superparamagnetic iron oxide nanoparticles^13^, ^14^. These iron oxide nanoparticles synthesized using the green approach demonstrated antiviral, antibacterial, antioxidative, anticancer, and anti-inflammatory properties^15^–^17^. Hence, in this study, we synthesized iron oxide nanoparticles by using the fruit extract of Pouteria caimito. The synthesized iron oxide nanoparticles were characterized using several techniques such as UV-Vis spectro, DLS, TEM, SEM-EDX, and FTIR.

UV-Vis spectro is the predominant technique used to preliminary analyze nanoparticle synthesis. UV-light absorption can be determined at various wavelengths to detect nanoparticle size and dispersal18. In our study, we examined synthesized PSNs through UV-Vis spectro at room temperature and measured absorbance at 277 and 844 nm; the results confirmed nanoparticle production.

TEM is used to determine particle size and distribution. Numerous studies have indicated that TEM clearly shows nanoparticles less than 100 nm in size. In our study, TEM findings showed that the PCSNs were nanorod shaped, with their size ranging from 9.41 to 16.96 nm (average size: 13.08 nm).

DLS is a unique, rapid, and nondestructive physical method used to evaluate the nanoparticles ranging from a few nanometers to few microns in size. Moreover, zeta potential is also measured to evaluate the electrostatic stability of nanoparticles. In this study, the PCSNs size distribution was observed at 186.6–847.3 d.nm with an average of 367.5 d.nm. The zeta potential analysis showed that PCSNs had uniform surface charge distribution, and its surface charge was equal to −13.7 mV. EDX is widely used for identifying the composition of organic molecules. Studies have reported that organic molecules overlapped with nanoparticles. Similarly, in our study, EDX results demonstrated that biomolecules present in nanoparticles synthesized using the fruit extract of Pouteria caimito fruit overlapped with iron nanoparticles because iron was the chief element. Furthermore, other biomolecules such as such as C, O, and Cl were identified, indicated the production of iron oxide nanoparticles.

FTIR is extensively used to identify different molecules and functional groups and determine the molecular structure. Recently, FTIR spectroscopy has been widely used in the field of nanotechnology for evaluating and identifying functional groups. The presence of a band at 3412 cm−1 indicated the existence of aliphatic compounds, and aromatic N-H stretching indicated the presence of amines. The band at 1629 cm−1 was associated with the carbonyl (C=C) group. Bands at 697 cm−1 and 471 cm−1 were related to Fe-O stretching. Furthermore, the band at 697 cm−1 indicated Fe-O-H stretching. The bands at 1075 cm−1 and 1384 cm−1 demonstrated the covalent attachment to a nanoparticle or ester. The band at 818 cm−1 was assigned to the C-C stretching of alkanes. Hence, our observed FTIR spectrum confirmed that Pouteria Caimito fruit can serve as an effective stabilizing and reducing agent during the synthesis of iron oxide nanoparticles.

The MTT assay is widely used to examine cytotoxicity because it is sensitive, easy to use, rapid, and low cost. In our study, we assessed the cytotoxic effects of PSN on MCF-7 and 3T3b cells. We examined the viability of cells after exposure to various concentrations of PSN. The results showed that the nanoparticles exerted minimal toxicity on both cell lines even at a higher concentration. Pouteria caimito is a common fruit and is rich in proteins, carbohydrates, fats, fibers, riboflavin, vitamin A, niacin, vitamin C, thiamine, Ca, and Fe8. In our study, we synthesized nanoparticles using this natural fruit. Hence, on the basis of cytotoxicity results, nanoparticles synthesized using this natural fruit can be used for drug delivery and cancer treatment. Moreover, their minimal toxicity at a higher dose can be vital for reducing side effects.

## Conclusion

The chemical and physical production of nanoparticles is a large-scale and high energy- consuming process. Numerous disadvantages occur during drug development such as the synthesis of hazardous byproducts and the production of organic toxic solvents. Hence, alternative biological and green methods are being developed because of their various advantages such as simplicity, nontoxicity, and specific target cell effectiveness. In our study, the Pouteria caimito fruit that is rich in vitamin A and C was successfully used as a bioreducer for the production of iron oxide nanoparticles. The Pouteria caimito fruit was determined to have a strong oxidizing nature and metal-reducing potential; thus, it is potentially attractive for the production of iron oxide nanoparticles. Moreover, the cytotxicity assay results revealed that iron oxide nanoparticles synthesized using the Pouteria caimito fruit extract derived can be used for targeting cancer cells and treating other diseases because of their nontoxic nature. These nanoparticles can be used for the treatment of cancer and other diseases in the future.
